# Impact of arabinoxylan-enriched diets on the intestinal chemical barrier and microbiota composition in rainbow trout (*Oncorhynchus mykiss*)

**DOI:** 10.3389/fvets.2024.1459001

**Published:** 2024-11-27

**Authors:** Xindang Zhang, Hengzhi Wang, Beibei Lin, Xiaolin Meng, Junming Deng

**Affiliations:** ^1^College of Fisheries, Henan Normal University, Xinxiang, China; ^2^College of Animal Science and Technology, Yunnan Agricultural University, Kunming, China; ^3^Tongwei Agricultural Development Co., Ltd., Chengdu, China; ^4^College of Fisheries, Guangdong Ocean University, Zhanjiang, China

**Keywords:** arabinoxylan, chemical barrier, intestinal microbiome, antioxidant function, rainbow trout

## Abstract

**Introduction:**

This study was conducted to evaluate the effects of dietary AX inclusion on the chemical barrier, antioxidant function and intestinal microbiome of rainbow trout.

**Methods:**

Five isoproteic and isolipidic experimental diets were formulated to contain 0.03% arabinoxylanase, as well as 0%, 2.5%, 5% and 10% AX (CAX, Con, AX2.5, AX5 and AX10), respectively.

**Results:**

The trypsin and maltase activities in the foregut of AX10 group were significantly lower than those in Con group. Similarly, the amylase and sucrase activities of the middle intestinal mucosa, maltase of the distal intestinal mucosa, and *MUC2* mRNA levels of the middle and distal intestinal mucosa in AX10 group were also lower than those in Con group. Additionally, the levels of GSH, GST, MDA in the plasma, SOD and CAT in the middle and distal intestinal mucosa, as well as MDA in the middle intestinal mucosa, were significantly higher in AX10 group compared to the CAX and Con groups. Conversely, the levels of CAT, GSH-Px, IGF-1, mTOR, AST in the plasma and AMPD, GDH in the liver were significantly lower in AX10 group compared to the CAX and Con groups. Furthermore, the Chao 1, Shannon index, and the abundance of Cyanobacteria, *Aurantimicrobium*, *Bacteroides* decreased with the decreasing dietary AX content. In contrast, the abundance of Proteobacteria, Actinobacteria, and *Stenotrophomonas* were increased in AX10 group compared to Con group.

**Discussion and conclusion:**

These results suggest that high AX (10%) diets may reduce the chemical barrier, antioxidant function, and protein metabolism in rainbow trout, while also reducing intestinal microbiome α-diversity and retarding the colonization of beneficial bacteria.

## Highlights

- Excessive dietary arabinoxylan (10%) damaged the chemical barrier of rainbow trout.
- Dietary 10% arabinoxylan reduced the antioxidant function of rainbow trout.
- Excessive dietary arabinoxylan retarded the colonization of beneficial bacteria in rainbow trout.

## 1 Introduction

For decades, the rapidly expanding aquaculture industry has driven a sharp increase in the demand for fish meal, making it a significant constraint on fishery resources (1, 2). Consequently, it is necessary to identify alternative protein sources for aquatic feed to mitigate the reliance on fish meal (3). Recently, plant proteins have emerged as a sustainable alternative to fish meal in aquatic feed formulations (4, 5). However, the extensive inclusion of plant proteins in diets can result in high levels of antinutritional factors, including phytic acid, tannic acid, non-starch polysaccharides (NSPs), and soybean agglutinin, which are detrimental to the growth and health of fish (6). Arabinoxylan (AX), a major class of NSP found in various cereals such as wheat, barley, rice, and maize, presents a particular challenge (7). Numerous studies in monogastric animals, including fish, indicate that they struggle to break down and digest AX in feed due to the absence of the necessary enzymes in their gastrointestinal tracts (8). Furthermore, AX’s high water-binding capacity can increase the viscosity of chyme, hindering the binding of nutrients and digestive enzymes and affecting the digestion and absorption of nutrients in the intestine (9, 10). Additionally, AX slows down digestion and reduces oxygen utilization, potentially creating a favorable environment for the growth of anaerobic bacteria and certain pathogens (11). As such, the anti-nutritional effects of AX may arise from its impact on intestinal digestive enzymes and the structure of the microbial community.

Intestinal mucosal chemical barrier mainly refers to mucopolysaccharides, digestive enzymes, active factors, and gastrointestinal hormones secreted by mucous cells within the mucous layer, which are closely related to the physiological functions of fish (12, 13). It resists the invasion of external harmful substances through biochemical action and protects the stability of the intestinal environment (14). Among these, protease, amylase, and lipase are the most common digestive enzymes, which participate in the decomposition of proteins, carbohydrates, and lipids, respectively (15). Additionally, the main components of mucus are mucins, a variety of glycosylated proteins synthesized and secreted by goblet cells (16). Mucoprotein 2 (MUC2) is a major mucin that can isolate bacteria and intestinal epithelial cells through its continuous renewal and supplementation, thereby defending against bacteria invasion of the intestinal epithelial cells and playing a barrier function (17). Further, studies have shown that the synthesis and secretion of MUC2 are affected by microorganisms and their metabolites (18). The adjustment of the intestinal microbiota crucial for the growth and health of the host, as it may be involved in the animal’s immune system, nutrient digestion and absorption, vitamin synthesis, the production of short-chain fatty acids, and the control of pathogen growth (19, 20). However, the physiological effects of dietary AX on fish intestinal microbiota, whether beneficial or harmful, remain controversial (21). For example, AX extracted from *Plantago asiatica* has been shown to alleviate type 2 diabetes by modulating the gut microbiota and its metabolites, while AX from corn bran has been reported to alter the composition of the bacterial community and decrease α-diversity (21, 22). This discrepancy may be due to the differences in AX structure, which lead to varying physiological functions and impacts on gut microbiota. Unfortunately, there are limited studies that focus on these tissues in fish.

As a carnivorous fish, the rainbow trout (*Oncorhynchus mykiss*) has emerged as one of the primary cold-water fish species bred in China, with an annual output of 30,000 tons (23). Several previous studies have demonstrated the adverse effects of dietary NSPs, such as cellulose and guar gum, on the growth performance and digestibility of rainbow trout (24, 25). Furthermore, our previous research within our group has indicated that the inclusion of 2.5–10% AX in the diet increases intestinal mucosal permeability, triggers an intestinal inflammatory response, and consequently impairs the intestinal mucosal barrier function and growth performance of rainbow trout (26). However, to date, no studies have confirmed the impact of AX on the intestinal chemical barrier and microbial community structure in rainbow trout. Therefore, the primary objective of this study is to investigate the effects of AX on intestinal digestive enzymes, antioxidant activity, and microbial community structure in rainbow trout, with the aim of elucidating the mechanisms by which dietary AX modulates the intestinal microbiota to influence intestinal health in carnivorous fish.

## 2 Materials and methods

### 2.1 Experimental diets

The four isoproteic and isolipidic experimental diets were supplemented with 0, 2.5, 5 and 10% AX (Con, AX2.5, AX5 and AX10). In addition, 300 mg/kg arabinoxylanase (800,000 U/g) was supplemented in the Con diet (CAX). The ingredients and proximate composition of the experimental diet was shown in Table 1. The experimental ingredients were ground through a 320-μm mesh sieve. All ingredients were thoroughly mixed with soybean oil and soybean lecithin, and the mixture was pelleted by an experimental pellet feed mill (KS-180; Jiangsu Jingu Rice Mill Co., Ltd., Jiangsu, China) through a 1.5-mm diameter die. The moist pellets were dried at 40°C for about 12 h in room temperature, and then stored at −20°C until used (26).

**Table 1:** Ingredients and proximate composition of experimental diets (%, dry matter basis).

Ingredients	CAX	Con	AX2.5	AX5	AX10
Fish meal^1^	50.00	50.00	50.00	50.00	50.00
Rapeseed meal^1^	5.00	5.00	5.00	5.00	5.00
Soybean meal^1^	5.50	5.50	5.50	5.50	5.50
Corn gluten meal^1^	3.00	3.00	3.00	3.00	3.00
Wheat starch	21.12	21.15	18.65	16.15	11.15
Arabinoxylan^2^	0.00	0.00	2.50	5.00	10.00
Arabinoxylanase^3^	0.03	0.00	0.00	0.00	0.00
Soybean oil	12.00	12.00	12.00	12.00	12.00
Vitamin premix^4^	1.00	1.00	1.00	1.00	1.00
Mineral premix^4^	0.50	0.50	0.50	0.50	0.50
Others^5^	1.85	1.85	1.85	1.85	1.85
Proximate composition					
Dry matter (DM, %)	94.47	94.52	93.93	93.08	93.48
Crude protein (% DM)	40.55	40.42	40.27	40.62	40.37
Crude lipid (% DM)	17.13	17.05	17.15	17.15	17.11
Ash (% DM)	14.15	14.35	14.44	14.37	14.33
Total energy (MJ/kg DM)	20.44	20.34	20.71	20.59	20.50
Soluble arabinoxylan (% DM)	0.37	0.39	2.82	5.11	9.87
Total arabinoxylan (% DM)	1.10	1.12	3.57	5.88	10.62
Viscosity (cP)	12.00	12.00	12.50	13.00	14.00

1 Supplied by Yunnan Xinhaifeng Food Co., Ltd. (Kunming, China): fish meal, crude protein, 67.59%, crude lipid, 7.72%; rapeseed meal, crude protein, 36.63%, crude lipid, 11.64%; soybean meal, crude protein, 46.27%, crude lipid, 0.73%; corn gluten meal, crude protein, 67.63%, crude lipid, 1.55%.

2 Supplied by Kunming Saijie Biotechnology Co., Ltd. (Kunming, China).

3 Supplied by Guangdong VTR Bio-Tech Co., Ltd. (Zhuhai, China).

4 Vitamin and mineral premix were supplied by Kunming Tongwei Co., Ltd. (Kunming, China). Vitamins premix (g kg^−1^): vit. A, 2; vit. D3, 0.03; vit. E, 30; vit. K3, 3; aneurine hydrochloride, 8; vit. B2, 11; vit. B6, 8; vit. B12, 0.02; vit. C, 50; folic acid, 1; biotin, 0.1; niacin, 30; calcium panate, 32; Inositol, 25. Mineral mixture (g kg^−1^): MgSO_4_·7H_2_O, 180; KI, 1; FeSO_4_·H_2_O, 260; ZnSO_4_·H_2_O, 180; CuSO_4_·5H_2_O, 25; Na_2_Se_2_O_3_, 0.01; MnSO_4_·H2O, 180; CoCl_2_·6H_2_O, 0.75.

5 Others including soybean lecithin, 0.5%; Ca(H_2_PO_4_)_2_, 1.0%; L-ascorbate-2-polyphosphate (35%), 0.02%; ethoxyquin (30%), 0.03%; choline chloride (50%), 0.3%.

### 2.2 Fish and experimental procedure

Rainbow trout (*Oncorhynchus mykiss*) obtained from a local commercial farm was acclimatized to the experimental conditions for 2 weeks before the start of the experiment. At the start of the feeding trial, healthy fish with similar sizes (initial average weight 3.14 ± 0.02 g) were distributed into 15 tanks (1.0 m × 0.7 m × 0.8 m) with 35 juveniles per tank. Each tank was then randomly assigned to one of three replicates of the five dietary treatments. Water was recirculated through biological and mechanical filters utilizing high-density polyester screens to remove particulate material and provide substrate for nitrifying bacteria. Fish were hand-fed to apparent satiation twice (07:00 and 17:00) daily for 9 weeks. During the feeding experiment, the water quality parameters were recorded as follows: temperature ranged from 16°C to 18°C, dissolved oxygen 7.8–9.2 mg/L, pH 6.7–7.7, total ammonia nitrogen 0.04–0.07 mg/L, and nitrite 0.02–0.04 mg/L.

### 2.3 Sample collection

At the end of the feeding trial, the fish were fasted for 24 h before harvesting. The experimental fish were anesthetized with eugenol (1:12,000; Shanghai Reagent Corporation, Shanghai, China) before sampling. A total of six fish per tank were randomly collected from each tank, and samples of plasma, liver, intestine, and intestinal mucosa were collected. Blood was collected from the caudal vein using a heparinised syringe and transferred into a heparinised tube. Plasma was recovered after centrifugation (4,000 g, 10 min) at 4°C and immediately stored at −80°C until analysis. The intestines of six fish per tank were quickly removed, then segmented into proximal (the section from the pyloric sphincter to the last pyloric cecum, PI), middle (the section distal to the most distal pyloric cecum and proximal to the increase in intestinal diameter, MI) and distal intestine (the section from the distal end of the MI to the anus, DI). The mucosa of PI, MI and DI were scraped from the serosa and stored frozen in liquid nitrogen and then stored at −80°C for subsequent determination. Another six fish were randomly collected from each tank for intestinal contents.

Dissections were performed on a low temperature sterile bench to avoid sample contamination. The whole intestinal contents of two fish in each cage were mixed to form a sample, collected in a 2-mL storage tube, and quickly frozen in liquid nitrogen and then transferred to −80°C for storage.

### 2.4 Chemical analysis

#### 2.4.1 Proximate composition

The proximate composition of feed ingredients and experimental diets were subjected following standard methods of AOAC: dry matter by drying to constant weight at 105°C; crude protein (*N* × 6.25) by the regular Kjeldahl method; crude lipid by the Soxhlet method with ether extraction; ash by incineration at 550°C for 16 h; gross energy in a bomb calorimeter (Parr1351; Parr Instrument Co., Moline, IL, United States). The total arabinoxylan contents were determined by the colorimetric method (70). For dietary viscosity measurement, 10 g crushed samples were mixed with 40 mL of distilled water, incubated for 30 min at 28°C, and then centrifuged at 12,000 × g for 10 min. The viscosity of the supernatant was immediately measured by NDJ-8S digital viscometer (Shanghai Precision & Scientific Instrument Co., Ltd., Shanghai, China), absolute viscosity was expressed in centipoise (cP) at a shear rate of 750/s.

#### 2.4.2 Intestinal mucosal chemical barrier-related parameters

The supernatant was obtained from the sample treated with reference to Deng et al. (26) as the enzyme source. The cholecystokinin (CCK) level in the foregut was measured using commercial ELISA kits for fish (Shanghai Enzyme Link Biotechnology Co., Ltd., Shanghai, China) according to the manufacturer’s protocols. Trypsin, lactase, maltase, sucrase, amylase and lysozyme activities were detected using commercial assay kits (Nanjing Jiancheng Bioengineering Institute, Nanjing, China) according to the manufacturer’s instructions (No. A080-2-2, No. A082-1-1, No. A082-3-1, No. A082-2-1, No. C016-1-1, No. A050-1-1). Specific enzyme activities were expressed as enzyme activity per gram soluble protein. The soluble protein content of extracts was determined according to Bradford (27).

#### 2.4.3 *MUC2* gene expression

Total RNA was extracted from intestinal (proximal, middle and distal) mucosa with RNAiso Plus (TaKaRa Biotechnology (Dalian) Co. Ltd., Dalian, China) (No. 9108), the concentration and integrity were determined by a nanophotometer (Nano Photometer NP80 Touch, Implen, Berlin, Germany) and 1.0% agarose gels. The qualified RNA samples were prepared to cDNA by an Evo M-MLV reverse transcription kit [TaKaRa Biotechnology (Dalian) Co. Ltd., Dalian, China] (No. 2680) and stored at −20°C for further use. The *MUC2* qPCR primers (Table 2) were designed using Primer 5.0 software based on the gene sequences in GenBank. The 18s was selected as the internal standard. The degrees of gene expression were identified by RT-qPCR (Bio-Rad, Richmond, CA, United States). The expressions of the target genes were determined by the $2^{-\Delta \Delta C_{T}}$ method (28).

**Table 2:** Forward and reverse primers for real-time quantitative PCR.

Name	Primer sequence (5′–3′)		NCBI Accession Number	Length (bp)	Temperature (°C)
*MUC2*	Forward	CCAGGCACAGAAAAGACAGATGC	XM_036968563.1	23	62.9
	Reverse	GGATGTAGGAGTGCTTGACC		20	
*18S*	Forward	TAACGAACGAGACTCCGGCA	NC_048575.1	20	62.8
	Reverse	GTTCATCGGGTTACCCACGC		20	

MUC2, mucoprotein 2.

#### 2.4.4 Antioxidant-related parameters

Plasma, middle, distal intestinal mucosa glutathione (GSH), superoxide dismutase (SOD), catalase (CAT), glutathione S-transferase (GST), malondialdehyde (MDA) contents and plasma glutathione peroxidase (GSH-Px), middle, distal intestinal mucosa reactive oxygen species (ROS) contents were measured by commercial kits (Nanjing Jiancheng Bioengineering Institute, Nanjing, China) (No. A006-2-1, No. A001-3-2, No. A007-2-1, No. A004-1-1, No. A003-1-2, No. A007-2-1, No. E004-1-1). The protein content in the microsomes was assayed according to the method described by Bradford (27).

#### 2.4.5 Protein metabolism-related parameters

The plasma and hepatic mammalian target of rapamycin (mTOR), glutamate dehydrogenase (GDH), adenosine monophosphate deaminase (AMPD) level and plasma growth hormone (GH), insulin growth factor-1 (IGF-1) level were measured using commercial ELISA kits for fish (Shanghai Enzyme Link Biotechnology Co., Ltd., Shanghai, China) according to the manufacturer’s protocols (No. ml001595, No. ml001447, No. ml261272, No. ml028539, No. ml025861). The aspartate aminotransferase (AST) and alanine aminotransferase (ALT) activities in plasma and hepatic were determined by using commercially available kits (Nanjing Jiancheng Bioengineering Institute, Nanjing, China) according to the manufacturer’s protocols (No. C010-1-1, No. C009-1-1).

#### 2.4.6 Intestinal flora analysis

The fresh intestinal contents collected were transported to Beijing Novogene Bioinformation Technology Co., Ltd. (Beijing, China), and total DNA was extracted. The 16S rRNA gene comprising V3–V4 regions was amplified using common primer pair and the microbial diversity analysis was performed. Briefly, the raw sequences were first quality-controlled using QIIME with default parameters, then demultiplexed and clustered into species-level (97% similarity) operational taxonomic units (OTUs). Taxonomic annotation analysis was obtained and the community composition of each sample was counted at each classification level using the Mothur method and the SSUrRNA database of Silva. Strain alpha diversity analysis and beta diversity analysis were performed using QIIME. In addition, the Venn diagram was drawn based on the data processed by homogenization and LEfSe analysis was used to identify species with significant differences between the groups.

### 2.5 Statistical analysis

All data were analyzed using one-way ANOVA, followed by a Tukey’s multiple range test. The significance level was set to *p* < 0.05. Statistical analysis was carried out using the SPSS 16.0 (SPSS Inc., Chicago, IL, United States) for Windows.

## 3 Results

### 3.1 Intestinal mucosal chemical barrier

The activities of foregut trypsin and maltase generally decreased with increasing dietary AX content. Notably, the trypsin and CCK activities in the AX10 group was significantly lower than that in other groups (*p* < 0.05; Table 3), and the maltase activity in the AX10 group was significantly lower than in the CAX group. There were no significant differences in the activities of LZM, lactase, and sucrase in the foregut among the different diets.

**Table 3:** The chemical barrier-related parameters in the intestinal contents, foregut and proximal, middle and distal intestinal mucosa of rainbow trout fed diets with different arabinoxylan content.

	CAX	Con	AX2.5	AX5	AX10	*p*-value
Intestinal contents						
pH	7.60 ± 0.09	7.63 ± 0.08	7.64 ± 0.09	7.83 ± 0.01	7.46 ± 0.11	0.950
Foregut						
CCK (ng/g protein)	17.58 ± 1.34^a^	20.26 ± 1.42^ab^	19.53 ± 1.58^ab^	19.43 ± 0.58^ab^	23.17 ± 1.74^b^	0.048
LZM (μg/mg protein)	0.14 ± 0.01	0.16 ± 0.02	0.14 ± 0.01	0.21 ± 0.01	0.19 ± 0.04	0.182
Trypsin (U/g protein)	38.47 ± 2.16^b^	58.09 ± 8.29^b^	47.55 ± 4.16^b^	45.96 ± 5.48^b^	8.92 ± 0.77^a^	0.002
Lactase (U/mg protein)	4.31 ± 0.88	4.53 ± 1.48	3.97 ± 0.51	3.00 ± 0.63	4.17 ± 2.03	0.917
Maltase (U/mg protein)	33.10 ± 4.35^b^	26.42 ± 3.11^ab^	26.83 ± 4.32^ab^	22.93 ± 3.58^ab^	13.91 ± 1.04^a^	0.033
Sucrase (U/mg protein)	40.62 ± 3.92	31.83 ± 7.72	29.05 ± 5.08	30.32 ± 5.61	35.66 ± 3.42	0.572
Proximal intestinal mucosa						
Trypsin (U/g protein)	10.35 ± 1.83	10.34 ± 1.09	10.20 ± 0.67	9.12 ± 0.38	7.93 ± 0.56	0.438
Amylase (U/mg protein)	5.55 ± 1.10	5.03 ± 0.62	5.36 ± 0.67	5.76 ± 0.62	5.48 ± 0.99	0.977
Lactase (U/g protein)	0.23 ± 0.05	0.16 ± 0.02	0.16 ± 0.04	0.23 ± 0.05	0.20 ± 0.02	0.522
Maltase (U/g protein)	0.97 ± 0.06	1.87 ± 0.39	1.97 ± 0.19	0.96 ± 0.07	1.48 ± 0.73	0.250
Sucrase (U/mg protein)	59.25 ± 7.55^a^	90.33 ± 9.56^ab^	101.21 ± 6.84^b^	84.05 ± 8.29^ab^	77.39 ± 10.96^ab^	0.042
Middle intestinal mucosa						
Trypsin (U/g protein)	9.23 ± 1.17	10.53 ± 0.56	9.04 ± 0.10	7.80 ± 0.68	7.35 ± 0.00	0.054
Amylase (U/mg protein)	8.90 ± 1.44^a^	18.15 ± 1.13^b^	13.59 ± 1.40^ab^	12.06 ± 1.06^a^	11.91 ± 0.70^a^	0.003
Lactase (U/mg protein)	98.49 ± 5.96	110.59 ± 4.41	80.87 ± 5.61	94.67 ± 10.35	82.15 ± 3.65	0.064
Maltase (U/g protein)	0.11 ± 0.01^a^	0.16 ± 0.01^b^	0.11 ± 0.01^a^	0.12 ± 0.00^a^	0.09 ± 0.01^a^	0.002
Sucrase (U/mg protein)	26.27 ± 1.75^a^	72.60 ± 8.26^c^	53.21 ± 2.84^bc^	48.51 ± 7.33^ab^	31.11 ± 0.13^ab^	0.001
Distal intestinal mucosa						
Trypsin (U/g protein)	7.08 ± 0.93	6.84 ± 1.55	7.89 ± 0.61	6.66 ± 0.24	7.29 ± 1.11	0.916
Amylase (U/mg protein)	2.20 ± 0.12	2.30 ± 0.13	2.22 ± 0.16	2.19 ± 0.09	2.28 ± 0.12	0.954
Lactase (U/g protein)	0.14 ± 0.01^b^	0.09 ± 0.00^a^	0.13 ± 0.01^ab^	0.13 ± 0.01^ab^	0.12 ± 0.01^ab^	0.021
Maltase (U/g protein)	0.10 ± 0.01^ab^	0.11 ± 0.01^b^	0.08 ± 0.01^ab^	0.08 ± 0.01^ab^	0.07 ± 0.00^a^	0.040
Sucrase (U/g protein)	0.07 ± 0.01^a^	0.16 ± 0.02^b^	0.17 ± 0.02^b^	0.15 ± 0.01^ab^	0.14 ± 0.02^ab^	0.014

^a,b,c^Values are presented as means ± SE (*n* = 3). Means in the same row with different superscripts are significantly different (*p* < 0.05).

Sucrase activity in the proximal intestinal mucosa exhibited a trend of initially increasing and then decreasing with increasing dietary AX content. The AX2.5 group showed the highest sucrase activity (*p* < 0.05; Table 3). There were no significant differences in the activities of trypsin, amylase, lactase, and maltase in the distal intestinal mucosa among the different diets.

The activities of amylase, maltase, and sucrase in the middle intestinal mucosa generally decreased with increasing dietary AX content. Particularly, the activities of amylase and sucrase in the Con group were significantly higher than those in the AX5 and AX10 groups (*p* < 0.05; Table 3). Maltase activity in the Con group was significantly higher than that in the AX2.5, AX5, and AX10 groups. No significant differences in the activities of trypsin and lactase were observed in the middle intestinal mucosa among the different diets.

Maltase activity in the distal intestinal mucosa of the Con group was significantly higher than that in the AX10 group (*p* < 0.05; Table 3). Lactase activity in the CAX group was significantly higher than that in the Con group. Conversely, sucrase activity in the CAX group was significantly lower than that in the C and AX2.5 groups. No significant differences in the trypsin and amylase activities of the distal intestinal mucosa were found among the different diets.

### 3.2 *MUC2* gene expression

**Figure 1:**
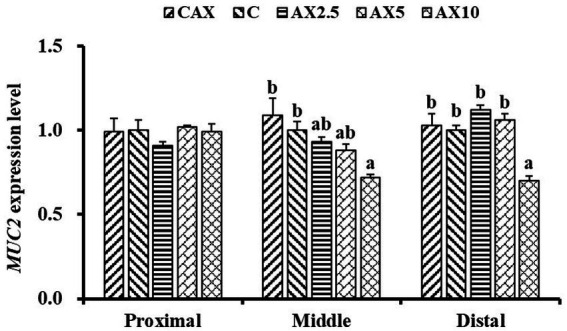
The *MUC2* (MUC2, mucoprotein 2) mRNA expression level in the proximal intestinal, middle intestinal and distal intestinal mucosa of rainbow trout fed diets with different arabinoxylan content. Values are means with their standard errors represented by vertical bars (*n* = 9). ^a,b^Bars with different superscripts are significantly different (*p* < 0.05).

In the middle and distal intestinal mucosa, the mRNA expression levels of *MUC2* decreased with the increasing dietary AX content (*p* < 0.05; Figure 1). The *MUC2* mRNA level in the middle intestinal mucosa was significantly lower in fish fed the AX10 diet compared to those fed the CAX and Con diets. Similarly, the *MUC2* mRNA level in the distal intestinal mucosa was significantly lower in fish fed the AX10 diet compared to those fed the other diets. However, no significant changes were found in the mRNA levels of *MUC2* in the proximal intestinal mucosa (*p* > 0.05).

### 3.3 Intestinal antioxidant capacity

Dietary inclusion of different levels of AX did not significantly affect the plasma SOD activity, middle intestinal mucosa GSH, GST, and ROS concentrations, or distal intestinal mucosa MDA and ROS concentrations (*p* > 0.05; Table 4). However, the plasma GSH, GST, and MDA concentrations in the AX10 group were significantly higher than those in the CAX and Con groups (*p* < 0.05). Conversely, the plasma CAT and GSH-Px concentrations in the AX10 group were significantly lower than those in the CAX and Con groups.

**Table 4:** The antioxidant function-related parameters in the plasma, and middle, distal intestinal of rainbow trout fed diets with different arabinoxylan content.

	CAX	Con	AX2.5	AX5	AX10	*p*-value
Plasma						
GSH (nmol/L)	0.06 ± 0.01^a^	0.07 ± 0.00^a^	0.11 ± 0.02^ab^	0.09 ± 0.01^ab^	0.14 ± 0.02^b^	0.025
SOD (U/mL)	12.85 ± 0.43	12.85 ± 0.29	11.96 ± 0.71	11.25 ± 0.80	11.99 ± 1.00	0.473
CAT (U/mL)	2.95 ± 0.06^b^	3.67 ± 0.21^b^	3.22 ± 0.64^b^	3.76 ± 0.47^b^	0.45 ± 0.09^a^	0.002
GST (U/mL)	57.04 ± 5.62^a^	44.52 ± 4.02^a^	41.28 ± 7.80^a^	47.30 ± 8.46^a^	86.72 ± 6.83^b^	0.018
GSH-Px (U/μL)	16.84 ± 2.11^b^	16.84 ± 2.11^b^	14.74 ± 2.11^ab^	8.42 ± 2.11^a^	8.42 ± 2.11^a^	0.027
MDA (nmol/mL)	8.99 ± 1.94^a^	5.58 ± 0.31^a^	6.11 ± 0.48^a^	9.07 ± 2.1^a^	21.01 ± 4.42^b^	0.005
Middle intestinal mucosa						
GSH (μmol/g protein)	0.06 ± 0.01	0.06 ± 0.02	0.06 ± 0.01	0.07 ± 0.01	0.05 ± 0.01	0.652
SOD (U/mg protein)	66.29 ± 3.73^a^	72.68 ± 1.99^a^	67.17 ± 4.92^a^	69.98 ± 4.30^a^	104.55 ± 6.66^b^	0.001
CAT (U/g protein)	4.55 ± 0.63^a^	4.46 ± 0.33^a^	5.35 ± 0.94^ab^	5.85 ± 0.18^ab^	7.18 ± 0.39^b^	0.036
GST (U/mg protein)	32.02 ± 3.17	35.89 ± 3.80	35.78 ± 5.22	36.86 ± 3.54	42.54 ± 2.00	0.428
MDA (nmol/mg protein)	5.77 ± 0.81^ab^	5.56 ± 1.19^a^	7.05 ± 1.44^ab^	8.49 ± 1.78^ab^	11.88 ± 1.21^b^	0.039
ROS (IU/μg protein)	0.12 ± 0.01	0.11 ± 0.01	0.12 ± 0.01	0.12 ± 0.01	0.14 ± 0.01	0.376
Distal intestinal mucosa						
GSH (μmol/g protein)	0.14 ± 0.01^a^	0.12 ± 0.00^a^	0.09 ± 0.02^a^	0.11 ± 0.01^a^	0.22 ± 0.02^b^	0.001
SOD (U/mg protein)	53.92 ± 1.19^a^	58.60 ± 2.42^a^	62.75 ± 5.67^a^	59.18 ± 4.16^a^	94.99 ± 8.20^b^	0.001
CAT (U/g protein)	5.05 ± 0.61^a^	4.92 ± 0.60^a^	6.49 ± 0.40^ab^	5.23 ± 0.27^a^	8.06 ± 0.89^b^	0.016
GST (U/mg protein)	97.08 ± 5.93^b^	66.88 ± 3.87^a^	83.60 ± 2.95^ab^	101.28 ± 4.97^b^	129.23 ± 9.13^c^	0.003
MDA (nmol/mg protein)	9.22 ± 1.46	9.14 ± 1.25	9.41 ± 1.27	10.13 ± 1.84	13.16 ± 0.83	0.266
ROS (IU/μg protein)	0.15 ± 0.01	0.11 ± 0.02	0.13 ± 0.01	0.14 ± 0.01	0.15 ± 0.02	0.258

^a,b,c^Values are presented as means ± SE (*n* = 3). Means in the same row with different superscripts are significantly different (*p* < 0.05).

In the middle intestinal mucosa, the SOD activity in the AX10 group was significantly higher than that in the other groups (*p* < 0.05; Table 4). Similarly, the CAT activity in the AX10 group was significantly higher than that in the CAX and Con groups. However, the MDA content in the AX10 group was significantly higher than that in the Con group.

In the distal intestinal mucosa, the GSH, SOD, and GST activities in the AX10 group were significantly higher than those in the other groups (*p* < 0.05; Table 4). Similarly, the CAT activity in the AX10 group was significantly higher than that in the CAX, Con, and AX5 groups.

### 3.4 Protein metabolism-related parameters

Dietary inclusion of different levels of AX did not significantly affect plasma GH, GDH, and ALT activities (*p* > 0.05; Table 5). Plasma IGF-1 and mTOR concentrations in the AX10 group were significantly lower than those in the CAX and Con groups (*p* < 0.05). Conversely, plasma AMPD and AST concentrations in the AX10 group were significantly higher than those in the other groups.

**Table 5:** The protein metabolism-related parameters in the plasma and hepatic of rainbow trout fed diets with different arabinoxylan content.

	CAX	Con	AX2.5	AX5	AX10	*p*-value
Plasma						
GH (μg/L)	22.68 ± 0.25	26.41 ± 0.37	24.29 ± 1.06	25.05 ± 0.37	23.07 ± 1.54	0.073
IGF-1 (μg/L)	28.79 ± 0.83^b^	31.69 ± 1.49^b^	28.27 ± 0.96^ab^	26.56 ± 1.68^ab^	21.76 ± 2.06^a^	0.009
mTOR (pg/L)	1.51 ± 0.02^b^	1.48 ± 0.02^b^	1.45 ± 0.08^b^	1.26 ± 0.07^ab^	0.89 ± 0.19^a^	0.004
GDH (U/L)	6.44 ± 0.18	6.42 ± 0.23	5.46 ± 0.35	5.75 ± 0.18	6.15 ± 0.39	0.126
AMPD (U/L)	73.78 ± 3.84^a^	81.89 ± 2.05^a^	83.27 ± 6.02^a^	85.27 ± 4.73^a^	104.83 ± 1.87^b^	0.003
AST (U/L)	61.24 ± 3.32^a^	56.10 ± 6.23^a^	60.91 ± 3.93^a^	58.58 ± 4.73^a^	89.56 ± 2.01^b^	0.001
ALT (U/L)	3.22 ± 0.18	4.03 ± 0.09	3.70 ± 0.54	3.55 ± 0.32	3.82 ± 0.11	0.426
Hepatic						
AMPD (U/g protein)	11.03 ± 0.35^ab^	12.44 ± 1.12^b^	11.55 ± 0.94^ab^	9.21 ± 0.98^a^	8.80 ± 0.75^a^	0.043
mTOR (pg/g protein)	0.12 ± 0.01	0.13 ± 0.01	0.13 ± 0.01	0.11 ± 0.01	0.12 ± 0.01	0.503
GDH (U/g protein)	0.91 ± 0.05^ab^	0.96 ± 0.03^ab^	1.02 ± 0.07^b^	0.82 ± 0.04^a^	0.82 ± 0.04^a^	0.048
AST (U/g protein)	9.69 ± 0.95	11.14 ± 1.96	10.69 ± 1.53	10.99 ± 1.73	10.04 ± 0.61	0.940
ALT (U/g protein)	5.25 ± 0.17^a^	5.25 ± 0.66^a^	5.64 ± 0.47^a^	5.30 ± 1.06^a^	7.96 ± 0.34^b^	0.046

^a,b^Values are presented as means ± SE (*n* = 3). Means in the same row with different superscripts are significantly different (*p* < 0.05).

Dietary inclusion of different levels of AX did not significantly affect hepatic mTOR and AST activities (*p* > 0.05; Table 5). Hepatic AMPD concentration in the AX5 and AX10 groups was significantly lower than that in the Con group (*p* < 0.05). Similarly, hepatic GDH concentration in the AX5 and AX10 groups was significantly lower than that in the AX2.5 group. Conversely, hepatic ALT concentration in the AX10 group were significantly higher than that in the other groups.

### 3.5 Microbiota structure analysis and functional prediction

**Figure 2:**
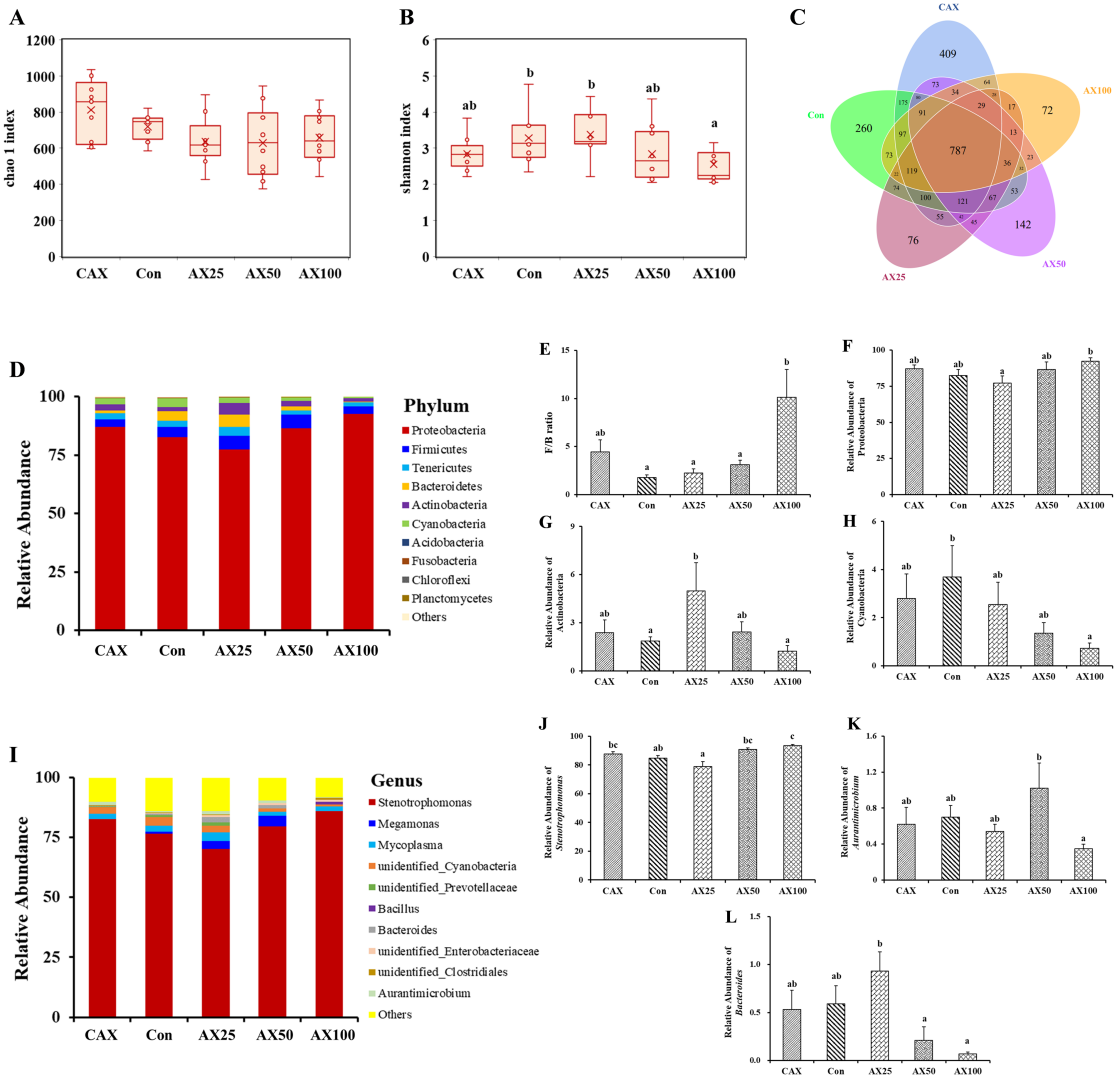
The effect of AX on the gut microbial diversity and composition. **(A)** Chao 1 index. **(B)** Shannon index. **(C)** Venn analysis at OUT level of each group of samples. **(D)** Relative abundance of the bacterial phylum. **(E)** Ratio of Firmicutes to Bacteroidetes. Relative abundance of Proteobacteria **(F)**, Actinobacteria **(G)**, Cyanobacteria **(H)**. **(I)** Relative abundance of bacteria in the top 10 genera. Relative abundance of *Stenotrophomonas* **(J)**, *Aurantimicrobium* **(K)**, *Bacteroides* **(L)**. Values are means with their standard errors represented by vertical bars (*n* = 6). ^a,b^Bars with different superscripts are significantly different (*p* < 0.05).

A total of 348,747 effective reads were derived from the stool samples, with an average of 69,749.46 reads per sample. Alpha diversity was expressed as the Chao1 index (Figure 2A) and the Shannon index (Figure 2B), which together reflected the richness and diversity of microbial communities. The Chao 1 index in the CAX group was significantly higher than that in the AX supplemental groups (AX2.5, AX5, and AX10) (*p* < 0.05). Additionally, the Shannon index in the Con and AX2.5 groups was significantly higher than that in the AX10 group (*p* < 0.05). Venn diagrams showed that the CAX, Con, AX2.5, AX5, and AX10 groups contained 409, 260, 76, 142, and 72 taxa in the OTUs, respectively (Figure 2C). Of all OTUs detected, 787 were commonly present in all five groups.

At the phylum level, the gut microbiota was primarily dominated by Proteobacteria, Firmicutes, Tenericutes, and Bacteroidetes (Figure 2D). The results showed that supplementation of 10% AX in the diet significantly increased the ratio of Firmicutes to Bacteroidetes (F/B) (*p* > 0.05; Figure 2E). Similarly, the relative abundance of Proteobacteria was significantly increased in the AX10 group (*p* < 0.05; Figure 2F). The relative abundance of Actinobacteria in the AX2.5 group was significantly higher than that in the Con and AX10 groups (*p* < 0.05; Figure 2G). In addition, the relative abundance of Cyanobacteria decreased as the dietary AX content increased (*p* < 0.05; Figure 2H).

At the genus level, the gut microbiota was primarily dominated by *Stenotrophomonas*, *Megamonas*, and *Mycoplasma* (Figure 2I). In addition, the abundance of *Stenotrophomonas* was significantly lower in the AX2.5 group compared to the AX10 group (*p* < 0.05; Figure 2J). Similarly, the abundance of *Aurantimicrobium* was significantly higher in the AX5 group than that in the AX10 group (*p* < 0.05; Figure 2K). The abundance of *Bacteroides* was significantly higher in the AX2.5 group than that in both the AX5 and AX10 groups (*p* < 0.05; Figure 2L).

**Figure 3:**
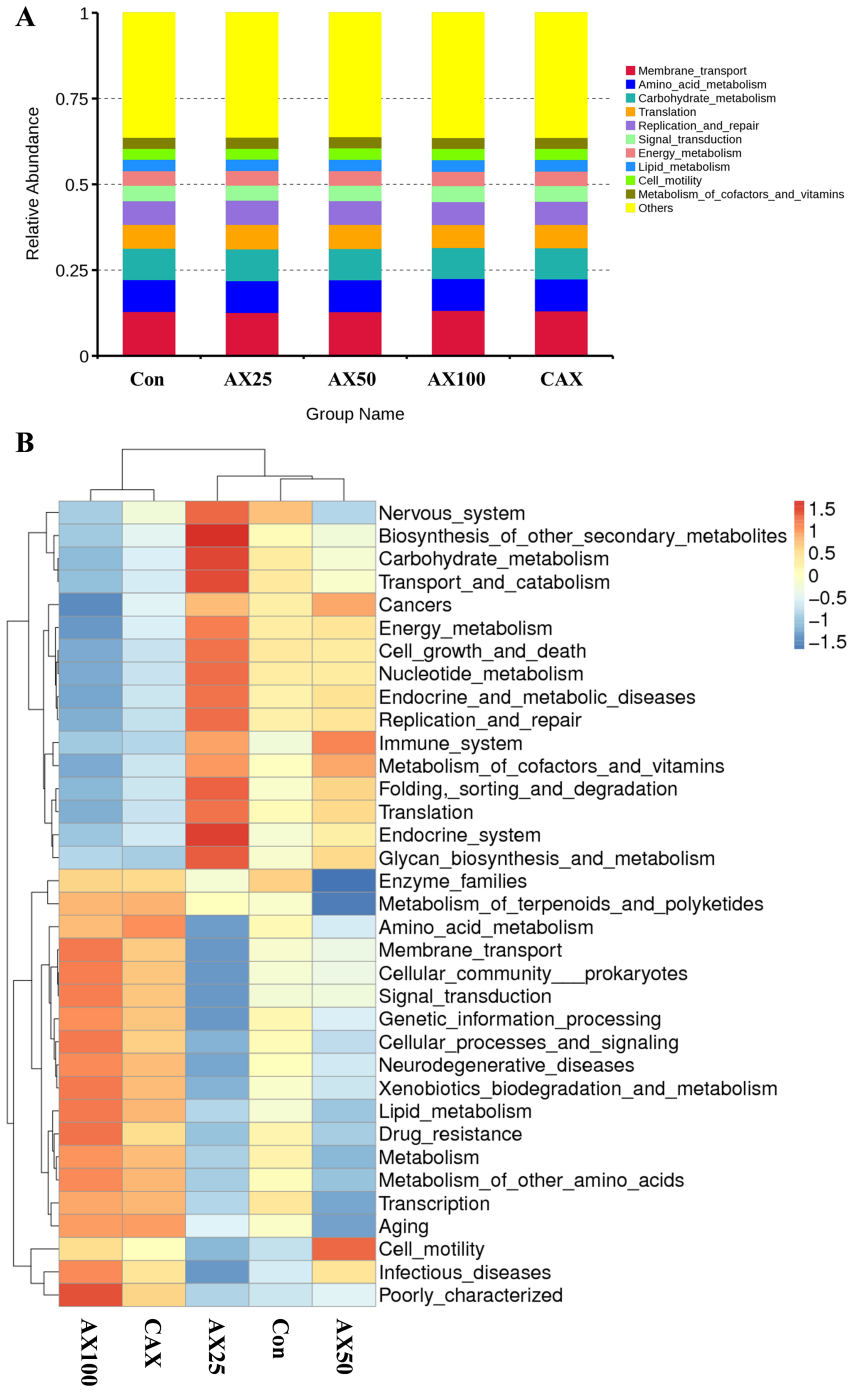
The function prediction of intestinal flora in rainbow trout fed with different AX diets. The predicted results of intestinal flora function are shown in **(A)**, and the clustering heatmap can more intuitively show the changes in intestinal flora function **(B)**.

The Tax4Fun tool was used to predict the function of the intestinal flora based on the analysis of effective reads (29), with the results presented in Figure 3A. Further, the cluster analysis more intuitively shows the changes in intestinal flora function (Figure 3B).

## 4 Discussion

This study aimed to investigate the effects of different levels of AX on the intestinal chemical barrier, antioxidant activity and microbial community structure of rainbow trout. The intestinal mucosal chemical barrier mainly refers to mucopolysaccharides, digestive enzymes, active factors, and gastrointestinal hormones secreted by mucous cells within the mucous layer, which are closely related to the physiological functions of fish (12, 13). Moreover, the intestine of fish is a complete organ that secretes digestive enzymes, which plays an indispensable role in digesting feed and absorbing nutrients (15, 30). Several reports have shown that the activity of digestive enzymes in fish intestines decreased from the proximal to the distal end (31). A similar downward trend in trypsin, lactase, and maltase activities was observed in rainbow trout, whereas no such tendency were found for amylase and sucrase activities in this study. Moreover, the results of this investigation showed that the activities of trypsin and maltase in the foregut were significantly decreased when supplemented with 10%AX, suggesting that excessive dietary AX could reduce fish digestion ability. Similarly, adding 16.8% soluble NSP to the diet significantly reduced the intestinal trypsin activity of rainbow trout (32). Moreover, it was also demonstrated that digestive enzymes activities were reduced in turbot fed high levels (5%) of soluble xylan (33). This indicated that higher levels of AX can create viscosity stress in the digesta, potentially damaging fish digestive organs and suppressing the secretion of digestive enzymes and other components (33, 34).

CCK is a widely distributed peptide in the gastrointestinal tract and central nervous system, which exhibits various physiological effects (35), including the stimulation of gallbladder contraction, gastric acid secretion, retardation of gastric emptying, and inhibition of energy intake (36). In this study, high dietary AX content (10%) significantly increased the foregut CCK concentration in rainbow trout, whereas no significant change was observed with low-dose dietary AX, suggesting that the effects of dietary AX on CCK secretion are dose-dependent. Several reports in mammals have shown that high levels of CCK can significantly reduce dietary intake by suppressing appetite and increasing satiety (37, 38). Additionally, MUC2 is the primary gel-forming and most abundant mucin in the intestine. It isolates bacteria and epithelial cells through its continuous renewal and secretion (17, 39). Furthermore, some studies have linked dietary intake of NSP to mucin concentrations in the gastrointestinal tract. For instance, the dietary addition of fiber or carboxymethyl cellulose increased ileal viscosity and mucin concentration in piglets (25), and dietary xylan at a concentration of 5% increased the expression of *MUC2* in the turbot intestine (40). However, excessive dietary AX (10%) significantly decreased the mRNA expression levels of *MUC2* in the middle and distal intestinal mucosa, while no significant change was observed with low-dose dietary AX (2.5–5%) in this study. These discordant findings may be attributed to differences in NSP species, as well as the structure and content of AX. These phenomena suggest that the poor growth of fish associated with a high AX diet may be related to the inhibitory effect of dietary AX on the intestinal chemical barrier.

It is well-known that antioxidant capacity encompasses enzymatic antioxidant activities (SOD, CAT, POD, GSH-Px) and non-enzymatic antioxidant contents (e.g., MDA) (41). Enzymatic antioxidants represent the body’s first line of defense against free radicals, while MDA is the final decomposition product of membrane lipid peroxidation and its levels can reflect the extent of stress-induced damage in fish (42). In the current study, we observed that the activities of GSH-Px, SOD, and CAT in the plasma of the 10% AX group decreased, and the MDA content increased, suggesting that dietary excessive AX induced oxidative stress in rainbow trout, leading to severe oxidative damage within the body. Similarly, soluble NSP has also been shown to induce oxidative stress in largemouth bass (43), rainbow trout (32), and tilapia (44). Furthermore, the activities of SOD and CAT in the middle and distal intestinal mucosa of the 10% AX group increased in our research, which may be attributed to the protective response of the intestinal against oxidative stress.

Furthermore, efficient protein synthesis requires the sufficient availability of all essential amino acids, and an imbalance in amino acids can result in decreased protein synthesis and increased protein degradation (45). Among these mechanisms, protein synthesis is dependent on insulin/IGF-1, TOR, and degradation on the ubiquitin-proteasome pathways, respectively (46). Additionally, when hepatocytes are severely necrotic or cell membrane permeability is increased, the content of AST/ALT distributed within hepatocytes rises, leading to elevated serum AST/ALT (47). Hence, the activities of IGF-1, mTOR, MAPD, GDH, AST, and ALT are considered critical markers for protein utilization and/or ammonia excretion (42). In this study, dietary excessive AX supplementation significantly reduced the levels of the protein synthesis indices mTOR and IGF-1 in rainbow trout plasma, while the content of the protein degradation index AMPD was significantly increased. This indicates that dietary excessive AX promotes protein decomposition and inhibits synthesis in rainbow trout, which would negatively impact growth performance. Furthermore, the AX10 group significantly increased plasma AST activity, suggesting that excessive AX caused liver function impairment in rainbow trout. Similarly, the inclusion of other soluble NSP in diets can also lead to liver function damage (32). For instance, dietary addition of 6% carboxymethyl cellulose severely damaged the liver in largemouth bass (43), and dietary addition of 15% pectin also severely damaged the liver in yellow catfish (48).

The intestinal is an important organ that has a huge surface area and allows for important interactions with microorganisms living within the intestine, known as the gut microbiome (19, 49). Additionally, the fish intestinal microbiome plays a crucial role in maintaining intestinal health, promoting intestinal development, resisting pathogen invasion, and facilitating nutrient digestion and absorption as well as lipid metabolism (40, 50, 51). Moreover, the formation and establishment of fish intestinal flora is complex, influenced by bacteria in dietary components and water environment (52, 53). Recently, the relationship between dietary NSP and the intestinal microbiome has garnered significant attention (54). In the present study, dietary supplementation with AX dramatically altered the Chao 1 and Shannon indices. More specifically, these indexes decreased with the increase of dietary AX content, and these were lowest in fish fed AX10 diet. In all the studies reviewed here, the Chao 1 and Shannon indices are used to reflect the richness and diversity of microbial communities (55). Healthy gut function requires microbial diversity, and a loss of diversity is associated with an increased risk of disease (49). Thus, these results suggest that excessive dietary AX tended to decrease the α-diversity of the intestinal flora in rainbow trout, also indicating that excessive dietary AX is unfavourable for intestinal flora homeostasis.

Dietary supplementation with AX significantly altered the intestinal microbial species abundance in rainbow trout. The dominant phylum groups of the intestinal flora were Proteobacteria, Firmicutes, Tenericutes, and Bacteroidetes, which was consistent with the findings of other studies on rainbow trout (56). This suggests that these phyla may constitute the core flora of rainbow trout. Although the addition of AX did not change the dominant phylum species in rainbow trout, it significantly altered the abundance of these dominant phyla. The ratio of *Firmicutes*/*Bacteroidetes* is widely considered to have an important effect on the maintenance of normal intestinal homeostasis, these varieties marked an ecological imbalance, which rised usually associated with obesity and reduced associated with inflammatory bowel disease (57). In the present study, the ratio of *Firmicutes*/*Bacteroidetes* was increased in the AX10 group. This indicates that dietary excessive AX may cause changes in the physiological function of the intestinal flora. Previous research has shown that Proteobacteria is a phylum sensitive to dietary intake and includes many pathogens (58), such as *Escherichia coli* and *Vibrio cholerae*, which have been recognized as potential markers of biological disorders and disease risk (59). Meanwhile, Actinomycete is a group of Gram-positive bacteria that produce bioactive secondary metabolites, which reduce the pathogenicity of pathogens (60). In addition, some studies have shown that Actinomycetes can reflect intestinal health to a certain extent, with higher abundance of Actinomycetes with better intestinal health (61). Furthermore, Cyanobacteria in the ocean are known for their nitrogen-fixing ability and production of antibacterial and antiviral natural products (62). In the present study, dietary excessive AX (10%) significantly increased the abundance of Proteobacteria, while the abundance of Actinobacteria and Cyanobacteria were decreased in the AX10 group. These findings indicate that dietary excessive AX could lead to an disorders in the structure of the rainbow trout intestinal flora.

Further research revealed that *Stenotrophomonas*, *Megamonas*, and *Mycoplasma* were the dominant genera in the intestine of rainbow trout across all groups in this study. *Stenotrophomonas* is a Gram-negative bacterium, with *Stenotrophomonas maltophilia* being an opportunistic pathogen that poses a high risk of morbidity and mortality in humans with compromised immune systems (63). In recent years, there have been reports both domestically and internationally of this bacterium causing illness or death in various mammals (64), and it has been identified in African catfish (65), channel catfish (66), and the West African dwarf crocodile (67). In this study, dietary supplementation with excessive AX (10%) significantly increased the abundance of *Stenotrophomonas*, which may help to explain the observed significant increase in the abundance of Proteobacteria in the AX10 group. Furthermore, dietary supplementation with excessive AX at 5 and 10% levels significantly decreased the abundance of *Bacteroides*. *Bacteroides* are capable of not only degrading feed proteins but also participating in carbohydrate metabolism and producing short-chain fatty acids (68), which not only provide energy for the host but also play a crucial role in mitigating the adverse effects of intestinal pathogens on host metabolism (69). The reduction in the abundance of *Bacteroides* may be one of the factors contributing to AX-induced enteritis in rainbow trout; however, this hypothesis requires further investigation (26). Collectively, these findings suggest that excessive AX (10%) may lead to an increase in the abundance of pathogenic microorganisms and a decrease in the abundance of beneficial bacteria, which is detrimental to the nutritional metabolism of rainbow trout.

## 5 Conclusion

The present results suggest that dietary low-dose arabinoxylan (<5%) had no significant negative effects on the intestinal chemical barrier and microbial community structure of rainbow trout. However, excessive dietary arabinoxylan (10%) exerted adverse effects on the intestinal chemical barrier by reducing intestinal digestive enzyme activities and *MUC2* gene expression, as well as simultaneous alterations in the abundance of Proteobacteria, Actinomycete, Cyanobacteria, and *Stenotrophomonas*, *Bacteroides*, which are associated with intestinal diseases. Furthermore, dietary 10% arabinoxylan also reduced the antioxidant function and protein metabolism of rainbow trout. Therefore, this study not only supplements and perfects the antinutritional mechanism of AX but also allows for the adjustment of the rainbow trout feed formula based on the results of this study, achieving efficient utilization of plant-based feed ingredients.

## Data Availability

The original contributions presented in the study are included in the article/supplementary material, further inquiries can be directed to the corresponding authors.

## References

[1] Hussain SM Bano AA Ali S Rizwan M Adrees M Zahoor AF. Substitution of fishmeal: highlights of potential plant protein sources for aquaculture sustainability. Heliyon. (2024) 10:e26573. doi: 10.1016/j.heliyon.2024.e26573, PMID: 38434023 PMC10906437

[2] Zhang Y Chen P Liang XF Han J Wu XF Yang YH. Metabolic disorder induces fatty liver in Japanese seabass, *Lateolabrax japonicas* fed a full plant protein diet and regulated by cAMP-JNK/NF-kB-caspase signal pathway. Fish Shellfish Immunol. (2019) 90:223–34. doi: 10.1016/j.fsi.2019.04.060, PMID: 31029777

[3] Zheng JC Zhang WC Dan ZJ Zhuang YW Liu YT Mai KS. Replacement of dietary fish meal with *Clostridium autoethanogenum* meal on growth performance, intestinal amino acids transporters, protein metabolism and hepatic lipid metabolism of juvenile turbot (*Scophthalmus maximus* L.). Front Physiol. (2022) 13:981750. doi: 10.3389/fphys.2022.981750, PMID: 36091361 PMC9451173

[4] Hua K Cobcroft JM Cole A Condon K Jerry DR Mangott A. The future of aquatic protein: implications for protein sources in aquaculture diets. One Earth. (2019) 1:316–29. doi: 10.1016/j.oneear.2019.10.018

[5] Tazikeh T Abedian KA Esmaeili M. Effects of fish meal replacement by meat and bone meal supplemented with garlic (*Allium sativum*) powder on biological indices, feeding, muscle composition, fatty acid and amino acid profiles of whiteleg shrimp (*Litopenaeus vannamei*). Aquac Res. (2020) 51:674–86. doi: 10.1111/are.14416

[6] He G Zhang T Zhou X Liu X Sun H Chen Y. Effects of cottonseed protein concentrate on growth performance, hepatic function and intestinal health in juvenile largemouth bass, *Micropterus salmoides*. Aquac Rep. (2022) 23:101052. doi: 10.1016/j.aqrep.2022.101052

[7] Luo S He L Zhang H Li Z Liu C Chen T. Arabinoxylan from rice bran protects mice against high-fat diet-induced obesity and metabolic inflammation by modulating gut microbiota and short-chain fatty acids. Food Funct. (2022) 13:7707–19. doi: 10.1039/d2fo00569g, PMID: 35758533

[8] Tiwari UP Fleming SA Rasheed MSA Jha R Dilger RN. The role of oligosaccharides and polysaccharides of xylan and mannan in gut health of monogastric animals. J Nutr Sci. (2020) 9:e21. doi: 10.1017/jns.2020.14, PMID: 32595966 PMC7303790

[9] Dalsgaard J Bach Knudsen KE Verlhac V Ekmann KS Pedersen PB. Supplementing enzymes to extruded, soybean-based diet improves breakdown of non-starch polysaccharides in rainbow trout (*Oncorhynchus mykiss*). Aquac Nutr. (2016) 22:419–26. doi: 10.1111/anu.12258

[10] Haidar MN Petie M Heinsbroek LTN Verreth JAJ Schrama JW. The effect of type of carbohydrate (starch vs. nonstarch polysaccharides) on nutrients digestibility, energy retention and maintenance requirements in Nile tilapia. Aquaculture. (2016) 463:241–7. doi: 10.1016/j.aquaculture.2016.05.036

[11] Stephan JG Khalid S Lourens W Neill JG. Effects of dietary supplementation of endo-(1,4)-β-xylanase in plant-based diets on growth performance, hindgut microbial diversity, and blood chemistry in large on-growing African catfish (*Clarias gariepinus*). J Appl Aquac. (2023) 35:561–84. doi: 10.1080/10454438.2021.2000920

[12] Rahimnejad S Lu K Wang L Song K Mai K Davis DA. Replacement of fish meal with *Bacillus pumillus* SE5 and *Pseudozyma aphidis* ZR1 fermented soybean meal in diets for Japanese seabass (*Lateolabrax japonicus*). Fish Shellfish Immunol. (2019) 84:987–97. doi: 10.1016/j.fsi.2018.11.009, PMID: 30403972

[13] Yu Z Xu S Zhao J Zhao L Zhang A Li M. Toxic effects of hexavalent chromium (Cr^6+^) on bioaccumulation, apoptosis, oxidative damage and inflammatory response in *Channa asiatica*. Environ Toxicol Phar. (2021) 87:103725. doi: 10.1016/j.etap.2021.103725, PMID: 34416396

[14] Li G Yuan H Fu Z Luo X Xue Z Zhang S. Investigating the impact of varied dietary protein levels on *Litopenaeus vannamei*: an exploration of the intestinal microbiota and transcriptome responses. Animals. (2024) 14:372. doi: 10.3390/ani14030372, PMID: 38338015 PMC10854741

[15] Yu Z Wu XQ Zheng LJ Dai ZY Wu LF. Effect of acute exposure to ammonia and BFT alterations on *Rhynchocypris lagowski*: digestive enzyme, inflammation response, oxidative stress and immunological parameters. Environ Toxicol Phar. (2020) 78:103380. doi: 10.1016/j.etap.2020.103380, PMID: 32416163

[16] Villarejo CP García AM Qian S Jiménez GS Domínguez PV Vélez PJF. Under the hood: understanding the features of mucin in pseudomyxoma peritonei. J Clin Med. (2023) 12:4007. doi: 10.3390/jcm12124007, PMID: 37373701 PMC10298939

[17] Bergstrom KS Kissoon SV Gibson DL Ma C Montero M Sham HP. Muc 2 protects against lethal infectious colitis by disassociating pathogenic and commensal bacteria from the colonic mucosa. PLoS Pathog. (2010) 6:e1000902. doi: 10.1371/journal.ppat.1000902, PMID: 20485566 PMC2869315

[18] Cornick S Tawiah A Chadee K. Roles and regulation of the mucus barrier in the gut. Tissue Barriers. (2015) 3:e982426. doi: 10.4161/21688370.2014.982426, PMID: 25838985 PMC4372027

[19] Dasriya VL Samtiya M Ranveer S Dhillon HS Devi N Sharma V. Modulation of gut-microbiota through probiotics and dietary interventions to improve host health. J Sci Food Agric. (2024) 104:6359–75. doi: 10.1002/jsfa.13370, PMID: 38334314

[20] Akhter N Wu B Memon AM Mohsin M. Probiotics and prebiotics associated with aquaculture: a review. Fish Shellfish Immunol. (2015) 45:733–41. doi: 10.1016/j.fsi.2015.05.038, PMID: 26044743

[21] Nie Q Hu J Chen H Geng F Nie S. Arabinoxylan ameliorates type 2 diabetes by regulating the gut microbiota and metabolites. Food Chem. (2022) 371:131106. doi: 10.1016/j.foodchem.2021.131106, PMID: 34543925

[22] Nguyen NK Deehan EC Zhang Z Jin M Baskota N Perez-Muñoz ME. Gut microbiota modulation with long-chain corn bran arabinoxylan in adults with overweight and obesity is linked to an individualized temporal increase in fecal propionate. Microbiome. (2020) 8:118. doi: 10.1186/s40168-020-00887-w, PMID: 32814582 PMC7439537

[23] Ma R Liu XH Meng YQ Wu JH Zhang L Han BY. Protein nutrition on sub-adult triploid rainbow trout (1): dietary requirement and effect on anti-oxidative capacity, protein digestion and absorption. Aquaculture. (2019) 507:428–34. doi: 10.1016/j.aquaculture.2019.03.069

[24] Storebakken T. Binders in fish feeds: I. Effect of alginate and guar gum on growth, digestibility, feed intake and passage through the gastrointestinal tract of rainbow trout. Aquaculture. (1985) 47:11–26. doi: 10.1016/0044-8486(85)90004-3

[25] Piel C Montagne L Sève B Lallès JP. Increasing digesta viscosity using carboxymethylcellulose in weaned piglets stimulates ileal goblet cell numbers and maturation. J Nutr. (2005) 135:86–91. doi: 10.1093/jn/135.1.86 15623838

[26] Deng JM Zhang XD Lin BB Mi HF Zhang L. Excessive dietary soluble arabinoxylan impairs the intestinal physical and immunological barriers via activating MAPK/NF-κB signaling pathway in rainbow trout (*Oncorhynchus mykiss*). Fish Shellfish Immunol. (2023) 141:109041. doi: 10.1016/j.fsi.2023.109041, PMID: 37657558

[27] Bradford MM. A rapid and sensitive method for the quantification of microgram quantities of proteins utilizing the principle of protein-dye binding. Anal Biochem. (1976) 72:248–54. doi: 10.1016/0003-2697(76)90527-3, PMID: 942051

[28] Livak KJ Schmittgen TD. Analysis of relative gene expression data using real-time quantitative PCR and the 2^-ΔΔ*C*_T_^ method. Methods. (2001) 25:402–8. doi: 10.1006/meth.2001.1262 11846609

[29] Aßhauer KP Wemheuer B Daniel R Meinicke P. Tax4Fun: predicting functional profiles from metagenomic 16S rRNA data. Bioinformatics. (2015) 31:2882–4. doi: 10.1093/bioinformatics/btv287, PMID: 25957349 PMC4547618

[30] Zhao J Yang L Jiang J Wu P Chen GF Jiang WD. Effects of dietary isoleucine on growth, the digestion and absorption capacity and gene expression in hepatopancreas and intestine of juvenile Jian carp (*Cyprinus carpio* var. Jian). Aquaculture. (2012) 368–369:117–28. doi: 10.1016/j.aquaculture.2012.09.019

[31] Mcleese JM Stevens ED. The effect of acclimation temperature, assay temperature, and ration on the specific activity of trypsin and chymotrypsin from rainbow trout (*Salmo gairdneri*). Comp Biochem. Physiol B. (1982) 73:631–4. doi: 10.1016/0305-0491(82)90087-6

[32] Deng J Zhang X Sun Y Mi H Zhang L. Effects of different types of non-starch polysaccharides on growth, digestive enzyme activity, intestinal barrier function and antioxidant activity of rainbow trout (*Oncorhynchus mykiss*). Aquac Rep. (2021) 21:100864. doi: 10.1016/j.aqrep.2021.100864

[33] Hu HB Mai KTN Zhang YJ Ai QH Xu W Zhang W. Effects of dietary xylan on growth performance, digestive enzyme activity and intestinal morphology of juvenile turbot (*Scophthalmus maximus* L.). Isr J Aquac - Bamidgeh. (2015) 67:1115. doi: 10.46989/001c.20730

[34] Sinha AK Kumar V Makkar HPS Boeck GD Becker K. Non-starch polysaccharides and their role in fish nutrition—a review. Food Chem. (2011) 127:1409–26. doi: 10.1016/j.foodchem.2011.02.042

[35] Asim M Wang H Waris A Qianqian G Chen X. Cholecystokinin neurotransmission in the central nervous system: insights into its role in health and disease. Biofactors. (2024) 22:1–16. doi: 10.1002/biof.2081, PMID: 38777339 PMC11627476

[36] Camilleri M. Gastrointestinal hormones and regulation of gastric emptying. Curr Opin Endocrinol Diabetes Obes. (2019) 26:3–10. doi: 10.1097/MED.0000000000000448, PMID: 30418188 PMC6615897

[37] Pathak V Flatt PR Irwin N. Cholecystokinin (CCK) and related adjunct peptide therapies for the treatment of obesity and type 2 diabetes. Peptides. (2018) 100:229–35. doi: 10.1016/j.peptides.2017.09.007, PMID: 29412823

[38] Hajishafiee M Ullrich SS Steinert RE Poppitt SD Luscombe-Marsh ND Horowitz M. Effects of intragastric tryptophan on acute changes in the plasma tryptophan/large neutral amino acids ratio and relationship with subsequent energy intake in lean and obese men. Food Funct. (2020) 11:7095–103. doi: 10.1039/d0fo00773k, PMID: 32729586

[39] Garrett WS Gordon JI Glimcher LH. Homeostasis and inflammation in the intestine. Cell. (2010) 140:859–70. doi: 10.1016/j.cell.2010.01.023, PMID: 20303876 PMC2845719

[40] Yang P Hu HB Li YX Ai QH Mai KS. Effect of dietary xylan on immune response, tight junction protein expression and bacterial community in the intestine of juvenile turbot (*Scophthalmus maximus* L.). Aquaculture. (2019) 512:734361. doi: 10.1016/j.aquaculture.2019.734361

[41] Cheng CH Guo ZX Ye CX Wang AL. Effect of dietary astaxanthin on the growth performance, non-specific immunity, and antioxidant capacity of pufferfish (*Takifugu obscurus*) under high temperature stress. Fish Physiol Biochem. (2018) 44:209–18. doi: 10.1007/s10695-017-0425-5, PMID: 28936571

[42] Zhang XD Zhang JW Wang HZ Lin BB Chen LS Li GB. Evaluation of soybean meal as alternative to fish meal in diet for juvenile Asian red tailed catfish (*Hemibagrus wyckioides*). Aquac Nutr. (2019) 25:1036–49. doi: 10.1111/anu.12921 PMC9973221 36860970

[43] Shi C Jiang Y Zhong Y Chen Y Luo L Lin S. Effects of dietary fiber sources on growth, plasma biochemical indexes, intestinal antioxidant capacity and histology of largemouth bass (*Micropterus salmoides*). J Fish China. (2019) 43:2485–93. doi: 10.11964/jfc.20190511814

[44] Jiang W Zhang Y Yuan M Liu Y Deng J Tan B. Effects of different types of non-starch polysaccharides on growth, digestive enzyme activity, intestinal barrier function and antioxidant activity of tilapia (*Oreochromis niloticus*). Aquac Rep. (2022) 25:101198. doi: 10.1016/j.aqrep.2022.101198

[45] Kumar S Zs JS Nagy Z Fazekas G Havasi M Sinha AK. Potential of processed animal protein versus soybean meal to replace fish meal in practical diets for European catfish (*Silurus glanis*): growth response and liver gene expression. Aquac Nutr. (2017) 23:1179–89. doi: 10.1111/anu.12487

[46] Syntichaki P Tavernarakis N. Signaling pathways regulating protein synthesis during ageing. Exp Gerontol. (2006) 41:1020–5. doi: 10.1016/j.exger.2006.05.014 16829008

[47] Talaveron JL Badía TMB Lozano AT Rigo BR Leiva BE. Phytosterolemia and gamma glutamyltransferase in adults with parenteral nutrition: fish versus vegetal lipids, randomized clinical trial. Nutrition. (2019) 70:1–19. doi: 10.1016/j.nut.2019.110587, PMID: 31743812

[48] Cai C Ren S Cui G Ni Q Cao X. Short-term stress due to dietary pectin induces cholestasis, and chronic stress induces hepatic steatosis and fibrosis in yellow catfish, *Pelteobagrus fulvidraco*. Aquaculture. (2019) 516:734607. doi: 10.1016/j.aquaculture.2019.734607

[49] Schupfer E Ooi SL Jeffries TC Wang S Micalos PS Pak SC. Changes in the human gut microbiome during dietary supplementation with modified rice bran arabinoxylan compound. Molecules. (2023) 28:5400. doi: 10.3390/molecules28145400, PMID: 37513272 PMC10385627

[50] Huang H Zhou P Chen P Xia L Hu S Yi G. Alteration of the gut microbiome and immune factors of grass carp infected with *Aeromonas veronii* and screening of an antagonistic bacterial strain (*Streptomyces flavotricini*). Microb Pathog. (2020) 143:104092. doi: 10.1016/j.micpath.2020.104092, PMID: 32145322

[51] Liu Y Cao Y Zhang Y Fan J Zhou H Huang H. Intestinal flora and immunity response to different viscous diets in juvenile largemouth bass, *Micropterus salmoides*. Fish Shellfish Immunol. (2022) 127:1012–23. doi: 10.1016/j.fsi.2022.06.054, PMID: 35863540

[52] Cryan JF Dinan TG. Mind-altering microorganisms: the impact of the gut microbiota on brain and behaviour. Nat Rev Neurosci. (2012) 13:701–12. doi: 10.1038/nrn3346, PMID: 22968153

[53] Meng XL Cai HM Li H You F Jiang AX Hu WP. *Clostridium butyricum*-fermented Chinese herbal medicine enhances the immunity by modulating the intestinal microflora of largemouth bass (*Micropterus salmoides*). Aquaculture. (2023) 562:738768. doi: 10.1016/j.aquaculture.2022.738768

[54] Liu Y Huang HJ Fan JT Zhou H Zhang YM Cao YX. Effects of dietary non-starch polysaccharides level on the growth, intestinal flora and intestinal health of juvenile largemouth bass *Micropterus salmoides*. Aquaculture. (2022) 557:738343. doi: 10.1016/j.aquaculture.2022.738343

[55] Forbes JD Van Domselaar G Bernstein CN. Microbiome survey of the inflamed and noninflamed gut at different compartments within the gastrointestinal tract of inflammatory bowel disease patients. Inflamm Bowel Dis. (2016) 22:817–25. doi: 10.1097/MIB.0000000000000684, PMID: 26937623

[56] Hodkovicova N Hollerova A Blahova J Mikula P Crhanova M Karasova D. Non-steroidal anti-inflammatory drugs caused an outbreak of inflammation and oxidative stress with changes in the gut microbiota in rainbow trout (*Oncorhynchus mykiss*). Sci Total Environ. (2022) 849:157921. doi: 10.1016/j.scitotenv.2022.157921 35952865

[57] Stojanov S Berlec A Trukelj B. The influence of probiotics on the Firmicutes/Bacteroidetes ratio in the treatment of obesity and inflammatory bowel disease. Microorganisms. (2020) 8:1715–30. doi: 10.3390/microorganisms8111715, PMID: 33139627 PMC7692443

[58] Xu Y Li Y Xue M Yang T Luo X Fan Y. Effects of dietary *Saccharomyces cerevisiae* YFI-SC2 on the growth performance, intestinal morphology, immune parameters, intestinal microbiota, and disease resistance of crayfish (*Procambarus clarkia*). Animals. (2021) 11:1963. doi: 10.3390/ani11071963, PMID: 34209070 PMC8300296

[59] Xie M Zhang S Xu L Wu Z Yuan J Chen X. Comparison of the intestinal microbiota during the different growth stages of red swamp crayfish (*Procambarus clarkii*). Front Microbiol. (2021) 12:696281. doi: 10.3389/fmicb.2021.696281, PMID: 34589066 PMC8473915

[60] Barka EA Vatsa P Sanchez L Gaveau-Vaillant N Jacquard C Meier-Kolthoff JP. Taxonomy, physiology, and natural products of *Actinobacteria*. Microbiol Mol Biol Rev. (2015) 80:1–43. doi: 10.1128/MMBR.00019-15, PMID: 26609051 PMC4711186

[61] Maier TV Lucio M Lee LH NC VB Brislawn CJ Bernhardt J. Impact of dietary resistant starch on the human gut microbiome, metaproteome, and metabolome. mBio. (2017) 8:e01343. doi: 10.1128/mBio.01343-17, PMID: 29042495 PMC5646248

[62] Niedermeyer TH. Anti-infective natural products from Cyanobacteria. Planta Med. (2015) 81:1309–25. doi: 10.1055/s-0035-1546055 26085049

[63] Brooke JS. Advances in the microbiology of *Stenotrophomonas maltophilia*. Clin Microbiol Rev. (2021) 34:e0003019. doi: 10.1128/CMR.00030-19, PMID: 34043457 PMC8262804

[64] Domosławska A Zduczyk S Jurczak A Janowski T. *Stenotrophomonas maltophilia* isolated from prostatic fluid as an infertility factor in a male dog. Andrologia. (2017) 49:e12769. doi: 10.1111/and.12769 28261845

[65] Abraham TJ Paul P Adikesavalu H Patra A Banerjee S. *Stenotrophomonas maltophilia* as an opportunistic pathogen in cultured African catfish *Clarias gariepinus* (Burchell, 1822). Aquaculture. (2016) 450:168–72. doi: 10.1016/j.aquaculture.2015.07.015

[66] Geng Y Wang K Chen D Huang X He M Yin Z. *Stenotrophomonas maltophilia*, an emerging opportunist pathogen for cultured channel catfish, *Ictalurus punctatus*, in China. Aquaculture. (2010) 308:132–5. doi: 10.1016/j.aquaculture.2010.08.032

[67] Harris NB Rogers DG. Septicemia associated with *Stenotrophomonas maltophilia* in a West African dwarf crocodile (*Osteolaemus tetraspis* subsp. *tetraspis*). J Vet Diagn Invest. (2001) 13:255–8. doi: 10.1177/104063870101300313, PMID: 11482606

[68] Power SE O’Toole PW Stanton C Ross RP Fitzgerald GF. Intestinal microbiota, diet and health. Br J Nutr. (2014) 111:387–402. doi: 10.1017/S0007114513002560 23931069

[69] Cui Y Zhang L Wang X Yi Y Shan Y Liu B. Roles of intestinal *Parabacteroides* in human health and diseases. FEMS Microbiol Lett. (2022) 369:72. doi: 10.1093/femsle/fnac072, PMID: 35945336

[70] Finnie SM Bettge AD Morris CF. Influence of cultivar and environment on water-soluble and water-insoluble arabinoxylans in soft wheat. Cereal Chem. (2006) 83:617–23. doi: 10.1094/CC-83-0617, PMID: 26937623

